# Synthetic surprise as the foundation of the psychedelic experience

**DOI:** 10.1016/j.neubiorev.2024.105538

**Published:** 2024-02

**Authors:** Roberto De Filippo, Dietmar Schmitz

**Affiliations:** a Charité-Universitätsmedizin Berlin, corporate member of Freie Universität Berlin, Humboldt-Universität Berlin, and Berlin Institute of Health, Neuroscience Research Center, 10117 Berlin, Germany; b German Center for Neurodegenerative Diseases (DZNE) Berlin, 10117 Berlin, Germany; c Charité-Universitätsmedizin Berlin, corporate member of Freie Universität Berlin, Humboldt-Universität Berlin, and Berlin Institute of Health, Einstein Center for Neuroscience, 10117 Berlin, Germany; d Charité-Universitätsmedizin Berlin, corporate member of Freie Universität Berlin, Humboldt-Universität Berlin, and Berlin Institute of Health, NeuroCure Cluster of Excellence, 10117 Berlin, Germany; e Humboldt-Universität zu Berlin, Bernstein Center for Computational Neuroscience, Philippstr. 13, 10115 Berlin, Germany

**Keywords:** Serotonin, 5-HT, 5-HT transcriptomics, Psychedelics, Predictive coding, Cognitive penetrability, Affective realism

## Abstract

Psychedelic agents, such as LSD and psilocybin, induce marked alterations in consciousness via activation of the 5-HT_2A_ receptor (5-HT_2A_Rs). We hypothesize that psychedelics enforce a state of synthetic surprise through the biased activation of the 5-HTRs system. This idea is informed by recent insights into the role of 5-HT in signaling surprise. The effects on consciousness, explained by the cognitive penetrability of perception, can be described within the predictive coding framework where surprise corresponds to prediction error, the mismatch between predictions and actual sensory input. Crucially, the precision afforded to the prediction error determines its effect on priors, enabling a dynamic interaction between top-down expectations and incoming sensory data. By integrating recent findings on predictive coding circuitry and 5-HT_2A_Rs transcriptomic data, we propose a biological implementation with emphasis on the role of inhibitory interneurons. Implications arise for the clinical use of psychedelics, which may rely primarily on their inherent capacity to induce surprise in order to disrupt maladaptive patterns.

## Highlights

• We review the literature supporting the notion that 5-HT signals surprise/prediction error.
• We posit that activation of 5-HT_2A_ receptors can elicit a synthetic surprise response.
• Synthetic surprise, via cognitive penetrability of perception and predictive coding, can explain the psychedelic state.
• We propose a possible biological implementation via activation of interneurons.
• We provide an online 5-HT_2A_ RNA visualizer, based on the mouse transcriptomic atlas from the Allen Institute.

## 1 Introduction

*“No account of the universe in its totality can be final which leaves these other forms of [altered] consciousness quite disregarded.“**William James (1902)*

The psychedelic experience, characterized by profound alterations in perception, cognition, and self-awareness, has captivated human curiosity for centuries (Nichols and Walter, 2021, George et al., 2022a, Schultes and Hofmann, 1980). From ancient rituals to modern scientific investigations, the exploration of altered states of consciousness through the use of psychedelics has been an intriguing and enduring topic (George et al., 2022b). Psychedelics, such as lysergic acid diethylamide (LSD), psilocybin, mescaline and N,N-dimethyltryptamine (DMT), have the ability to induce remarkable and often transformative subjective experiences (Nichols, 2016, Freedman, 1968, Timmermann et al., 2021, Barrett and Griffiths, 2018). The psychedelic experience can be described as a departure from ordinary waking consciousness. It leads to an altered state of awareness that transcends the boundaries of conventional perception and cognition. Participants often report vivid visual and auditory alterations, a noetic quality, synesthesia, profound shifts in emotions, a sense of awe, altered sense of time, and a heightened sense of connection with the self, others, and the surrounding environment (Barrett and Griffiths, 2018). Psychedelics seem to have the potential to unravel the fabric of our conventional reality, unveiling its deeply contingent and subjective nature.

The study of psychedelics and their effects on consciousness has experienced a resurgence in recent years, driven by the exploration of their potential clinical applications, after decades of limited research because of legal and regulatory restrictions. In this work we propose a new model to explain the effect of psychedelic compounds on cognitive functions informed by recent experimental observations linking 5-HT to surprise, prediction error and uncertainty. Three concepts inherently related to each other (Feldman and Friston, 2010). Activation of 5-HT_2A_Rs is universally recognized as the mechanism underlying the subjective experience provoked by psychedelics (Nichols, 2016, Glennon et al., 1984). According to our hypothesis the psychedelic state should be considered as an alteration of the native emotional state of surprise, which we refer to as synthetic surprise. Here the qualifier synthetic is used deliberately to underscore the significant distinctions in comparison to the natural surprise emotion.

Synthetic surprise is posited to emerge from the partial activation of the 5-HTR system, in contrast to the response observed under physiological conditions following the release of endogenous 5-HT. We can assume that native 5-HT activates all 5-HTRs according to specific binding affinities. Psychedelics deviate from this pattern by selectively activating only a subset of receptors. Moreover, in some cases, binding kinetics are remarkably different (Wacker et al., 2017, Kim et al., 2020). The activation of different patterns of 5-HTRs can certainly elicit profoundly different downstream effects. For example, 5-HT_1_R and 5-HT_2_R have opposing effect on the membrane potential, causing respectively inhibition and excitation (Araneda and Andrade, 1991) and can co-localize on the same neuron (Amargós-Bosch et al., 2004, Santana et al., 2004, Wedzony et al., 2008).

The proposed model is built upon two theoretical frameworks: the cognitive permeability of perception and the predictive coding hypothesis. These two concepts are interconnected, as cognitive permeability can be effectively understood and described within the framework of predictive coding (Lupyan, 2015). Notably, surprise, in the context of predictive coding, refers to the magnitude of the prediction error or the level of mismatch between the predicted and actual sensory input. Surprise plays a crucial role in the predictive coding framework as it drives learning and adaptation processes (Friston, 2010, Barrett and Simmons, 2015). The brain constantly refines its internal models to align with sensory inputs, thereby minimizing prediction errors. This adaptation improves understanding of the environment and the accuracy of future predictions.

**Fig. 1:**
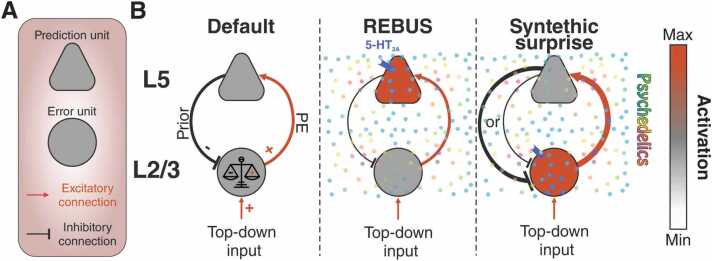
Different effects of REBUS and synthetic surprise models on a simplified predictive coding circuitry. A Components of a simplified predictive coding circuit, in which first order predictions are computed via subtraction. Second order predictions are not included. B According to a classical predictive coding implementation, L2/3 pyramidal neurons are responsible for computing the prediction error (PE) by comparing the top-down excitatory input with the inhibitory prior transmitted by deep layer (L5) pyramidal neurons. The REBUS model focus on the excitation of deep pyramidal neurons mediated by 5-HT_2A_Rs. This increase in excitability is not, however, strengthening the prior they encode, symbolized by the thinner black line, instead, it is changing the nature of how these neurons respond to incoming information, sensitizing them to prediction error signals arising from L2/3. The synthetic surprise model, on the contrary, considers activation of L2/3 error units the paramount effect induced by psychedelics. Precision associated to the prior (not depicted in the panel) is modulated independently and can be either over or underweighted (indicated by the lines with different weight). 5-HT_2A_Rs in purple.

Various models have been proposed to explain the effect of psychedelics. Some theories, like the cortico–striato–thalamocortical (Vollenweider and Geyer, 2001) and the cortico–claustro–cortical model (Doss et al., 2022), focus on implementation, highlighting altered circuits without placing significant emphasis on explaining the psychological effects. Other theories, like the strong prior hypothesis to explain hallucinations (Corlett et al., 2019), on the contrary focus on the psychological effects. The REBUS model (Carhart-Harris and Friston, 2019), built also on the foundation of predictive coding, attempts to provide explanations for both the psychological effects and the specific alterations in neural circuits induced by psychedelics. We can outline the synthetic surprise model against the backdrop of the REBUS model, given the similar foundation, as an alternative perspective with a notably different implementation. At the heart of the REBUS model is the concept that psychedelics exert their effects by diminishing the precision assigned to high-level beliefs or priors, thereby increasing the precision of prediction errors and facilitating the flow of bottom-up sensory information. In contrast, the synthetic surprise model posits an increase in the prediction error signal itself, rather than in its precision. This distinction has significant implications. A steady prediction error signal is anticipated to increase the expected uncertainty, therefore lowering the ability of the errors to influence priors, corresponding to a strengthening of priors. Accordingly, learning rates are known to decrease in the presence expected uncertainty (Preuschoff and Bossaerts, 2007, Bach and Dolan, 2012, Payzan-Lenestour et al., 2013, Soltani and Izquierdo, 2019). When priors are more precise than sensory data, they can potentially shape perception, aligning with the strong prior hypothesis for hallucinations (Powers et al., 2017, Corlett et al., 2019, Powers et al., 2016). The computing of precision, however, seems to be distributed among several neuromodulators including dopamine and acetylcholine (Feldman and Friston, 2010, Yu and Dayan, 2005, Moran et al., 2013, Pérez-González et al., 2023, Haarsma et al., 2021, Gershman, 2017, Soltani and Izquierdo, 2019). Notably, dopamine and acetylcholine have been historically associated with the neural regulation of selective attention (Noudoost and Moore, 2011), a process that has been hypothesized to optimize precision (Feldman and Friston, 2010, Smout et al., 2019, Friston, 2009). In summary, while the expected effect of a persistent artificial prediction error signal is to increase the precision afforded to priors, we do not exclude that the influence of other neuromodulators can, in some instances, increase the precision of sensory data, resulting in a relaxation of priors as described in the REBUS model. This interplay enables a dynamic interaction between top-down expectations and sensory information (Fig. 1).

The synthetic surprise model strives to offer a solution to certain discrepancies of the REBUS model. Specifically, the emphasis of the REBUS model on a reduction in the precision of top-level priors does not easily account for the enhanced "imaginative suggestibility" observed under psychedelics (Carhart-Harris et al., 2015, Sjoberg and Hollister, 1965, Middlefell, 1967, Leary, 1961). “Imaginative suggestibility” is the capacity of an individual to immerse themselves in imaginative scenarios that can influence their behavior and subjective experience. In a recent placebo-controlled study (Carhart-Harris et al., 2015), it was found that the perceived realism and vividness of imagined scenarios, following detailed auditory instructions, were significantly heightened under the influence of LSD. Complementing this finding, ethnographic studies often note a cultural consistency in hallucinatory experiences, supporting a culturalist perspective on psychedelic hallucinations (Dupuis, 2022a, Dupuis, 2022b). Factors beyond the drug itself, including mindset (expectations, preparation, intention) and environment (physical and social settings), significantly influence these hallucinogenic experiences (Dupuis, 2021, Dupuis, 2022a, Hartogsohn, 2017, Hartogsohn, 2016, Leary, 1961). To account for the influence of imaginative suggestions on perception, it seems logical to incorporate top-down control of perception (Terhune et al., 2017, Kosslyn et al., 2001). This mechanism allows individuals to align their perceptual experiences with the expectations that arise from external suggestions. It is not trivial to reconcile these observations with the main tenet of the REBUS model, a relaxation of the precision associated with top-level priors. Second, it is widely recognized that hallucinations can occur in healthy individuals under conditions of sensory deprivation, particularly in complete darkness (Vernon et al., 1961, Bexton et al., 1954, Scott et al., 1959, Cohen et al., 1959, Flynn, 1962, Vosburg et al., 1960). In such instances, where sensory input is limited or absent, the brain may generate its own perceptual experiences, resulting in hallucinations. This phenomenon highlights that vivid stimuli alone do not typically alter perceptions, but rather hallucinations can arise in the absence of external sensory stimulation, such as in complete darkness. Closed-eyes hallucinations are commonly reported under psychedelics (Carhart-Harris et al., 2016b) and it is unclear how a relaxation of priors can explain these phenomena (Kwan et al., 2022). Both discrepancies can theoretically be explained by abnormal precision assigned to priors.

At a neurophysiological level, the REBUS model places significant emphasis on the increased spiking activity of deep-layer pyramidal neurons induced by 5-HT_2A_R activation. If this were the primary effect of psychedelics, it would suggest a general enhancement of excitatory drive throughout the cortical network. However, the network effects of 5-HT_2A_R activation seems to be more intricate and nuanced, and they do not align with a simple increase in gain of excitatory cells (Kwan et al., 2022, Tyler et al., 2023). For instance, studies in rodents employing selective activation of 5-HT_2A_R through optogenetic (Eickelbeck et al., 2019) or pharmacological (Michaiel et al., 2019) stimulation consistently show a decrease in stimulus-evoked activity in the visual cortex. Furthermore, 5-HT_2A_Rs have been implicated in mediating divisive scaling of evoked responses, leading to a bidirectional modulation by suppressing neurons with strong responses and facilitating neurons with weak responses (Azimi et al., 2020, Shimegi et al., 2016, Watakabe et al., 2009). Further research, utilizing optogenetic stimulation and fMRI techniques in mice, has established a correlation between the inhibition of activity across the cortex, triggered by 5-HT release, and the expression of 5-HT_2A_R (Grandjean et al., 2019). Additionally, activation of 5-HT_2A_R in rats with psychedelics inhibited spontaneous population activity in a variety of cortical regions (Wood et al., 2012, Brys et al., 2023). Given that 5-HT_2A_R activation typically leads to depolarization and increased spiking, it is reasonable to assume that the reduction in activity of pyramidal neurons is mediated by the activation of inhibitory interneurons induced by 5-HT_2A_R. Inhibition or direct interneuron activation by 5-HT_2A_R has been reported across various brain structures in addition to visual cortex, including prefrontal cortex (PFC) (Abi-Saab et al., 1999, Ashby et al., 1990, Athilingam et al., 2017, Ashby et al., 1989), piriform cortex (Marek and Aghajanian, 1994, Sheldon and Aghajanian, 1990), cingulate cortex (Zhou and Hablitz, 1999), cochlear nucleus (Tang and Trussell, 2017), amygdala (Sengupta et al., 2017), olfactory bulb (Petzold et al., 2009, Hardy et al., 2005), entorhinal cortex (De Filippo et al., 2021) and hippocampus (Wyskiel and Andrade, 2016).

In summary, the synthetic surprise model, informed by recent experimental insights regarding 5-HT, differs from the REBUS model in two key aspects. First, it proposes that psychedelics induce primarily an artificial prediction error, with an impact contingent on its assigned precision. In this context hallucinations can be explained by the strong prior hypothesis (Corlett et al., 2019). Second, the biological implementation of the synthetic surprise model shifts its focus from excitatory to inhibitory cells.

We will start by briefly introducing the concepts of cognitive permeability of perception and the predictive coding hypothesis. Subsequently we will describe the psychedelic experience with its biological underpinnings, novel findings connecting 5-HT with the prediction error and our model with its implications.

## 2 Affect and cognitive penetrability of perception

Affect has been known to influence behavior and judgment since antiquity. Competition between reason and emotion has been a staple philosophical concept throughout history from Plato to Freud (Peters, 1970). The influence of emotions can be categorized as either *integral* or *incidental* (Lerner et al., 2015). Integral emotions are derived from the choice at hand, its potential outcomes and repercussions. While they were once viewed as detrimental to decision-making (Keltner and Lerner, 2010), current consensus sees integral emotions as a potential beneficial guide (Lerner et al., 2015) in line with the trailblazing view proposed by David Hume (Hume, 2003). Compelling scientific validation of this perspective comes from individuals afflicted by emotional impairments resulting from injuries to the ventromedial prefrontal cortex (vmPFC). This brain region plays a pivotal role in integrating cognition and emotion (Roy et al., 2012). Research shows that such neurological impairments significantly diminish both the capacity of patients to experience emotions and the effectiveness of their decision-making processes (Bechara et al., 1997, Abel et al., 2016, Sutterer et al., 2016, Waters-Wood et al., 2012, Schneider and Koenigs, 2017, Hiser and Koenigs, 2018). Studies show that participants with vmPFC injuries consistently exhibit a tendency to choose riskier financial options over safer ones, even to the extent of facing bankruptcy in real-money games (Clark et al., 2008). Remarkably, despite their cognitive understanding of the suboptimal nature of their choices, these individuals repeatedly make decisions that lead to detrimental outcomes. Physiological measurements, such as galvanic skin response, provide insights into the underlying reasons for this behavior. They suggest that the absence of emotional somatic markers prevents these individuals from experiencing reasonable fear in the face of high-risk scenarios (Bechara et al., 1997), which is typically present in normal decision-makers (Lerner et al., 2015).

Incidental emotions influence decisions by carrying over from unrelated situations, usually without awareness (Loewenstein and Lerner, 2003, Han et al., 2007, Bodenhausen, 1993). These affective states bias in a systematic normative judgment and decision-making. While a valence-based approach categorizes emotions into positive and negative, suggesting that good moods induce optimism and bad moods pessimism, this approach has its limitations. The Appraisal-Tendency Framework offers a multi-dimensional perspective, suggesting that emotions not only predispose individuals to specific behaviors but also to appraise their environment in particular ways (Lerner et al., 2015). However, biases in judgment can arise when these appraisals stem from incidental emotions. In line with this idea, incidental emotions have an influence on risk-taking (Ferrer and Ellis, 2021, Edmans et al., 2007, Hirshleifer and Shumway, 2003, Schulreich et al., 2014, Halko et al., 2015, Yang et al., 2020, Raghunathan and Pham, 1999, Han et al., 2007), perceived life-satisfaction (Schwarz and Clore, 1983), prosocial behavior (Knapp and Clark, 1991, Kassinove et al., 2002, Drouvelis and Grosskopf, 2016, Motro et al., 2016, Desteno et al., 2014, Bartlett and Desteno, 2006), food-choice and consumption (Garg et al., 2007, Wu et al., 2022, Evers et al., 2018).

One explanation for the effect of emotions or mood on behavior posits that affect, even when incidental, is an intrinsic facet of perception. In the same way as color is considered to be a property of an object, affect is also incorporated in the perception of reality (Wormwood et al., 2019). This hypothesis is called *affective realism* (Anderson et al., 2012, Barrett and Bar, 2009, Kring et al., 2014) and aligns with experimental data showing the impact of affect on perception (Zadra and Clore, 2011). Individuals experiencing negative affect exhibit perceptual biases such as heightened perception of sound intensity (Siegel and Stefanucci, 2011), increased sensitivity to visual contrast gradients (Phelps et al., 2006), and a propensity for local rather than global perceptual processing of images (Gasper and Clore, 2002). Furthermore, stimuli with negative valence are often perceived as larger in comparison to neutral or positive stimuli (Teachman et al., 2008, Van Ulzen et al., 2008), and individual differences in motivation and past experiences influence perception (Balcetis and Dunning, 2006). For instance, individuals experiencing unpleasant thirst perceive a glass of water as taller (Veltkamp et al., 2008), and individuals with spider phobia perceive spiders as larger (Leibovich et al., 2016). Affective realism can be considered a variant of the affect-as-information theory (Clore, 1992, Clore et al., 2001, Forgas, 1998, Forgas and George, 2001, Forgas, 1995) that states that individuals use their current affective state as a way to gain information from the environment, therefore, blurring the line between incidental and integral emotions (Ferrer and Ellis, 2021). Affective realism differentiates itself by explicitly positing affect as part of the conscious perception of an object or situation and not merely influencing the judgment process downstream from perception. Some experimental evidence supports this hypothesis, providing empirical validation for the integration of affect within the conscious perceptual experience (Wormwood et al., 2019, Siegel et al., 2018, Kraus and Hesselmann, 2023, Anderson et al., 2011).

Affective realism inherently assumes the cognitive penetrability of perception (Pylyshyn, 1980, Pylyshyn, 1999). Traditionally, cognition, perception, and action were viewed as entirely independent processes (Hurley, 1998, Pylyshyn, 1999, Firestone and Scholl, 2016). The validity of this perspective has been radically challenged by claims that perception should be considered as a way of acting, and not a purely passive event (O'regan and Noë, 2001, Vetter and Newen, 2014). Evidence for the top-down influence on visual perception stems from the organization of the brain inter-regional connectivity, the speed of temporal processing and functional experiments (Newen and Vetter, 2017). All visual areas are modulated by upstream inputs (Gilbert and Li, 2013, Markov et al., 2013), retina included (Schröder et al., 2020). Top-down influences originate from various sources, encompassing multiple areas that convey information in accordance with the functional properties specific to each area, these influences have the capacity to modify the information transmitted by neurons in two ways: by modifying the responsiveness of neurons to stimulus properties, or by altering the patterns of correlations within neuronal ensembles (Gilbert and Li, 2013). Various studies show that top-down influences on neuronal activity can occur in less than 50 ms after stimulus onset (Vetter et al., 2015, Wibral et al., 2009, Hupé et al., 2001). Collectively, these results demonstrate that the brain possesses the necessary infrastructure to enable the influence of cognition on low-level perception. The effect of expectation on perception has been investigated in some classic experiments showing a change in the perceived color of a stimulus object shaped by the expectations formed through prior interactions and familiarity (Delk and Fillenbaum, 1965, Bruner and Postman, 1949). More recent studies have confirmed these results (Hansen et al., 2006, Olkkonen et al., 2008), further pointing to a significant modulation of high-level visual memory on color perception. Even when objects are presented in an achromatic format, a discernible neural representation of the associated color can be decoded in the primary visual cortex V1 (Bannert and Bartels, 2013). Observed changes in perception were also linked to activation of areas not part of the early visual system supporting the interaction of perception with memory and attentional processes (Dolan et al., 1997). Importantly the effect of expectations and attention can be differentiated. Top-down influences seem to potentially modify expectations, which play a central role in perception (De Lange et al., 2018, Powers et al., 2016). Fulfilled expectations are associated to improved object recognition and reduced neural responses. Interestingly, the predictive coding theory offers a convincing theoretical framework to explain these observed effects (Summerfield and Egner, 2009, Friston, 2005).

## 3 Predictive coding hypothesis

The predictive coding hypothesis has gained significant traction in neuroscience as a compelling framework for understanding the mechanisms underlying perception and cognition. This hypothesis posits that the brain actively generates predictions about sensory inputs and uses these predictions to update and refine its internal models of the world (Von Helmholtz, 1867, Friston, 2005, Rao and Ballard, 1999, Craik, 1943). At its core, the predictive coding hypothesis is rooted in the principle of Bayesian inference. According to this perspective, the brain aims to minimize prediction errors by continuously updating its internal models based on the discrepancy between expected and actual sensory inputs (Lee and Mumford, 2003, Aitchison and Lengyel, 2017, Heeger, 2017). Furthermore, the concept of perceptual inference and the significance of prior expectations in shaping perception have deep historical roots, tracing back to the 11th century with the work of Ibn al-Haytham. He already recognized that the perception of many visible properties relies not merely on the external objects but also on the processes of judgment and inference (Sabra, 1989).

Although predictive coding encompasses a variety of interpretations (Spratling, 2017), our focus is on the predictive processing that juxtaposes sensory input with a generative environmental model. This comparison is understood to occur within a hierarchical structure, composed of multiple, successive systems that are involved in prediction and novelty detection (Wacongne et al., 2011, Caucheteux et al., 2023). Prediction errors are deeply tied to their precision or uncertainty. This relationship allows us to differentiate the magnitude of prediction errors from their reliability (Hohwy, 2013b). In the context of a noisy or volatile environment, the continuous signaling of large prediction errors may not necessarily lead to substantial updates of expectations, mainly due to the inherent imprecision of the prediction errors. Conversely, even slight deviations between sensory inputs and descending predictions can trigger significant updates of conditional expectations when the prediction errors exhibit high precision. The significance of precision lies in its ability to minimize surprise about the amplitude of prediction errors, a second order prediction. Precision can be manipulated externally, such as through alterations in the contrast or statistics of the stimulus, or internally, by directing attention to specific sensory streams or altering the contextual expectancy of sequential stimuli. Remarkably, attention plays a pivotal role in anticipating precise sensory information or prediction errors. The concept of precision gains particular importance in the interpretation of certain empirical findings, where repetition suppression can be influenced by contextual factors like attention (Auksztulewicz and Friston, 2016). The predictive coding hypothesis has profound implications for our understanding of perception and cognition. It posits that perception is an active process, where top-down predictions shape and guide the processing of bottom-up sensory inputs. This framework can account for various perceptual phenomena, including perceptual illusions and the influence of prior knowledge on perception (Lerer et al., 2021, Weilnhammer et al., 2017, Lumer et al., 1998).

Experimental evidence in human using functional neuroimaging has provided support for the predictive coding hypothesis across various sensory modalities (Walsh et al., 2020, Cao et al., 2019, Aitchison and Lengyel, 2017). Reports have shown that the brain's response to sensory stimuli is influenced by the magnitude of prediction errors. Larger prediction errors elicit stronger neural responses, indicating that the brain is sensitive to deviations from its predictions (Garrido et al., 2009, Todorovic et al., 2011, Wacongne et al., 2011, Meyer and Olson, 2011). While empirical evidence has strongly supported the concept of hierarchical inference in the cortex, the precise implementation by cortical neurons remains less understood. One theory posits the existence of error units that directly compare top-down and bottom-up information generating a prediction error signal (Bastos et al., 2012). When there is a mismatch or discrepancy between the top-down predictions and the incoming sensory inputs, a prediction error signal is generated. This prediction error signal represents the magnitude of the mismatch and serves as a feedback signal to update the existing predictions. The error units play a crucial role in adjusting and refining the predictions, facilitating the alignment between the internal model and the incoming sensory information. The theory suggests that prediction error signals are propagated hierarchically, with higher-level brain regions receiving feedback about the prediction errors and updating their predictions accordingly. This iterative process allows the brain to continually update its internal representations and improve the accuracy of its predictions (Mikulasch et al., 2023).

Early evidence supporting the predictive processing framework in neocortex came from studies on classical visual phenomena, such as end-stopping (Hubel and Wiesel, 1968). These phenomena can be explained as prediction errors, where the suppression of neuronal responses in the surround of a classical receptive field is attributed to top-down inhibition (Rao and Ballard, 1999). This concept of top-down prediction inhibiting bottom-up input has been used to explain various classical visual receptive field properties within the framework of predictive processing (Keller and Mrsic-Flogel, 2018). In the mouse visual cortex, SST interneurons, likely driven by lateral projections from neighboring cortical neurons, have been found to play a causal role in surround suppression (Adesnik et al., 2012, Keller et al., 2020). Surround suppression involves the inhibition of spiking in response to stimuli presented in the surrounding area of its receptive field. Traditionally, sensory neurons are characterized by tuning curves, which illustrate how their firing rates change in response to various stimulus attributes. In the case of surround suppression, the neuron's responses to stimuli outside the region that typically triggers firing are suppressed. While the majority of research on surround suppression has focused on visual cortex, it seems to be a widespread computation observed also in olfactory (Aungst et al., 2003), somatosensory (Sachdev et al., 2012) and auditory cortices (Gilday et al., 2023, Anna et al., 2020).

Recent experimental data investigating the effect of visuomotor mismatch seems to give support to the existence of error units in visual cortex. The effects of visual input and locomotion-related input on the membrane potential of neurons in the neocortex were found to be opposing in layer 2/3 (L2/3) neurons (Jordan and Keller, 2020). This observation supports the hypothesis that L2/3 neurons compute a difference between visual (bottom-up) and motor (top-down) inputs. In line with other reports showing prediction error signals in L2/3 (Muzzu and Saleem, 2021, Homann et al., 2022). In contrast, deep-layer neurons, usually associated with encoding of expectations (Bastos et al., 2012), did not exhibit this characteristic, showing depolarizing responses to both types of input. Moreover, different L2/3 excitatory neurons responded to the mismatch with significantly different change in membrane potential, ranging from strong depolarization to strong hyperpolarization. These responses align with signaling positive or negative prediction errors, which can be attributed to the opposing inputs from visual and locomotion-related sources. Importantly, this computational characteristic appears to be specific to L2/3 neurons. According to this predictive coding microcircuit, activity of positive and negative prediction error neurons should be anticorrelated, therefore when positive prediction neurons are excited negative prediction neurons should instead be inhibited (Jordan and Keller, 2020, Keller and Mrsic-Flogel, 2018). It is plausible to suggest that the differentiation between these two functional types of neurons in L2/3 is determined by variations in circuit wiring (Jordan and Keller, 2020). One element of the circuitry associated to negative prediction error L2/3 neurons has been elucidated: the visually driven inhibition onto these neurons originates from SST interneurons (Attinger et al., 2017), which selectively target the apical dendrites of L2/3 excitatory neurons (Rudy et al., 2011). Recently, a specific subset of SST interneurons, Sst44 cells, in the posterior parietal cortex was observed to activate synchronously during trajectory corrections for successful goal-directed navigation, effectively providing a navigation prediction error signal (Green et al., 2023). Interestingly, while Sst44 cells in the retrosplenial cortex also show synchronous activation, they do not convey navigation error signals. This suggests that the functional relationship between this subset of cells, and behavior or sensory inputs may be region-specific.

The role of SST interneurons in predictive coding is reflected by their responses to predictable and deviant stimuli. Inhibiting SST interneurons activity specifically diminishes the detection of deviations in cortical circuits, on the other hand, baseline responses remain largely unaffected at the cellular or circuit level (Hamm and Yuste, 2016). Conversely, predictable stimuli are associated with reduced activity of SST interneurons (Bastos et al., 2023, Heintz et al., 2022). However, contrasting findings have been reported, with some studies indicating that SST interneurons are more reactive to familiar rather than novel stimuli (Natan et al., 2017, Natan et al., 2015, Hayden et al., 2021, Kato et al., 2015, Arriaga and Han, 2019). Extensive recording in visual cortex using longitudinal 2-photon calcium reveals that SST interneurons can respond differently to novelty, either being inhibited or excited, thereby providing a reconciliation of these conflicting findings (Garrett et al., 2023). Stimulus adaptation upon repeated exposure can be understood as an expression of the brain's efficient strategy for minimizing prediction error by adapting its predictions concerning the content and precision of incoming sensory inputs (Auksztulewicz and Friston, 2016, Summerfield and De Lange, 2014). While these findings underscore the importance of SST interneurons in the predictive coding circuitry, the precise relationship between adaptation and different types of prediction errors remains unclear.

In summary, cortical SST interneurons are involved both in the modulation of surround suppression and in the computation of prediction errors, highlighting their potential significance in predictive coding processing.

## 4 Neuronal underpinnings of the psychedelic experience

As early as the 1950 s, it was hypothesized that the alteration of subjective experience caused by psychedelics was related to the 5-HT system. This pioneering idea stemmed from the observation that the chemical structures of LSD and 5-HT bear a striking resemblance (Woolley and Shaw, 1954). Soon after, it was proposed that LSD might act as an agonist of a specific class of 5-HTRs (Shaw and Woolley, 1956). Remarkably, this idea was advanced before the confirmation of the existence of different classes of 5-HTRs (Gaddum and Picarelli, 1957). Subsequently, chlorpromazine, a typical antipsychotic, was found to significantly dampen the psychological alterations induced by LSD in humans (Isbell and Logan, 1957). At the time this result was misattributed to the general sedative effect of the compound. Convincing evidence collected since those early years of psychedelic research have confirmed that psychedelics exert their overt effect on consciousness by interacting with the 5-HT system, and more precisely, by activating the 5-HT_2A_R. Chlorpromazine is indeed a potent 5-HT_2A_R antagonist (Trichard et al., 1998). The administration of ketanserin, a selective blocker of 5-HT_2A_R, can significantly reduce the subjective effects of several psychedelics in humans (Preller et al., 2017, Preller et al., 2018c, Kraehenmann et al., 2017a, Kraehenmann et al., 2017b, Vollenweider et al., 1998). Moreover, the “head twitch response” in rodents, a behavioral proxy for the effect of psychedelics (Halberstadt et al., 2020), was shown to rely on 5-HT_2A_R (Halberstadt, 2015, Keiser et al., 2009, González-Maeso et al., 2007). Studies in humans also indicate a correlation between the psychedelic effects of psilocybin and the occupancy of 5-HT_2A_Rs, as measured through positron emission tomography in cortical regions (Madsen et al., 2019). Furthermore, hallucinogenic potency is highly correlated to 5-HT_2A_R binding affinity (Glennon et al., 1984). While some psychedelics have a considerably large binding spectrum that includes 5-HT, dopamine, and adrenergic targets, it is clear that 5-HT_2A_R plays a mechanistically fundamental role in the psychedelic experience (Halberstadt and Geyer, 2011).

The activation of the 5-HT_2_R is associated with an elevation in intracellular calcium levels, resulting in the depolarization of the resting potential and possibly increased spiking activity (Nichols and Nichols, 2008). 5-HT_2_Rs are located throughout the cortical sheet, in the prefrontal, cingulate, visual, temporal and motor areas (Jakab and Goldman-Rakic, 1998). They are often found on the apical dendrites of pyramidal neurons (Jakab and Goldman-Rakic, 1998, Willins et al., 1997) but they are also present on inhibitory interneurons. This was shown using both immunohistochemistry (Jakab and Goldman-Rakic, 1998, Morilak et al., 1993, Morilak et al., 1994, Santana et al., 2004, De Filippo et al., 2021, Weber and Andrade, 2010) and electrophysiological methods (Gellman and Aghajanian, 1993, De Filippo et al., 2021, Marek and Aghajanian, 1996, Sheldon and Aghajanian, 1990). Given the localization on both excitatory and inhibitory neurons, the downstream effect of 5-HT_2_R agonism is difficult to predict.

Experimental findings regarding the influence of psychedelics on brain waves in human present a complex picture, with diverse outcomes reported across different studies (Table 1). While delta, gamma, and theta bands have shown both an increase (Timmermann et al., 2019, Pallavicini et al., 2021, Timmermann et al., 2023) or a decrease (Muthukumaraswamy et al., 2013, Carhart-Harris et al., 2016b, Murray et al., 2022, Schenberg et al., 2015) in either global or localized patterns, one consistent observation emerges: a pervasive decline in cortical alpha oscillations across psychedelics (Timmermann et al., 2023, Timmermann et al., 2019, Pallavicini et al., 2021, Muthukumaraswamy et al., 2013, Carhart-Harris et al., 2016b, Schenberg et al., 2015, Kometer et al., 2013, Murray et al., 2022, Eckernäs et al., 2023). In most cases this reduction in alpha power is positively correlated with the intensity of the subjective psychological effect. Functional imaging studies in humans have revealed also that psychedelics significantly impact the default mode network (DMN), comprising the medial prefrontal cortex, posterior cingulate cortex, and parietal regions (Andrews‐Hanna et al., 2014). This network is typically active during rest and internal mental processes. Psychedelics are found to decrease the functional connectivity within the DMN, the amount of covarying neural activity occurring simultaneously across different brain regions. Additionally, they increase connectivity between sensory regions belonging to different networks (Gattuso et al., 2023, Carhart-Harris et al., 2016b, Nichols, 2016, Tagliazucchi et al., 2016, Carhart-Harris et al., 2012, Preller et al., 2018b, Müller et al., 2018, Madsen et al., 2021). Neuroimaging studies combined with pharmacological blockade methods have confirmed that these LSD-induced changes in functional network configuration are dependent on the activation of 5-HT_2A_Rs (Preller et al., 2018a).

**Table 1:** Summary of Neuroimaging Studies on Psychedelic Substances.

**Year**	**Title**	**Substance**	**Measure**	**Band**	**Effect**	**Location**
2023	Human brain effects of DMT assessed via EEG-fMRI	DMT	EEG	Alpha	Decrease	Widespread
2019	Neural correlates of the DMT experience assessed with multivariate EEG	DMT	EEG	Alpha	Decrease	Widespread
2021	Neural and subjective effects of inhaled N,N-dimethyltryptamine in natural settings	DMT	EEG	Alpha	Decrease	Widespread
2013	Broadband cortical desynchronization underlies the human psychedelic state	Psylocibin	MEG	Alpha	Decrease	Widespread
2016	Neural correlates of the LSD experience revealed by multimodal neuroimaging	LSD	MEG	Alpha	Decrease	Widespread
2015	Acute Biphasic Effects of Ayahuasca	DMT	EEG	Alpha	Decrease	Localized
2013	Activation of Serotonin 2 A Receptors Underlies the Psilocybin-Induced Effects on α Oscillations, N170 Visual-Evoked Potentials, and Visual Hallucinations	Psylocibin	EEG	Alpha	Decrease	Widespread
2022	Low doses of LSD reduce broadband oscillatory power and modulate event-related potentials in healthy adults	LSD	EEG	Alpha	Decrease	Widespread
2023	N,N-dimethyltryptamine affects electroencephalography response in a concentration-dependent manner-A pharmacokinetic/pharmacodynamic analysis	DMT	EEG	Alpha	Decrease	Widespread
2019	Neural correlates of the DMT experience assessed with multivariate EEG	DMT	EEG	Beta	Decrease	Localized
2013	Broadband cortical desynchronization underlies the human psychedelic state	Psylocibin	MEG	Beta	Decrease	Widespread
2016	Neural correlates of the LSD experience revealed by multimodal neuroimaging	LSD	MEG	Beta	Decrease	Widespread
2022	Low doses of LSD reduce broadband oscillatory power and modulate event-related potentials in healthy adults	LSD	EEG	Beta	Decrease	Widespread
2013	Broadband cortical desynchronization underlies the human psychedelic state	Psylocibin	MEG	Delta	Decrease	Widespread
2016	Neural correlates of the LSD experience revealed by multimodal neuroimaging	LSD	MEG	Delta	Decrease	Widespread
2022	Low doses of LSD reduce broadband oscillatory power and modulate event-related potentials in healthy adults	LSD	EEG	Delta	Decrease	Widespread
2023	Human brain effects of DMT assessed via EEG-fMRi	DMT	EEG	Delta	Increase	Localized
2019	Neural correlates of the DMT experience assessed with multivariate EEG	DMT	EEG	Delta	Increase	Localized
2021	Neural and subjective effects of inhaled N,N-dimethyltryptamine in natural settings	DMT	EEG	Delta	Increase	Localized
2013	Broadband cortical desynchronization underlies the human psychedelic state	Psylocibin	MEG	Gamma	Decrease	Widespread
2015	Acute Biphasic Effects of Ayahuasca	DMT	EEG	Gamma	Decrease	Localized
2022	Low doses of LSD reduce broadband oscillatory power and modulate event-related potentials in healthy adults	LSD	EEG	Gamma	Decrease	Widespread
2023	Human brain effects of DMT assessed via EEG-fMRI	DMT	EEG	Gamma	Increase	Localized
2021	Neural and subjective effects of inhaled N,N-dimethyltryptamine in natural settings	DMT	EEG	Gamma	Increase	Localized
2013	Broadband cortical desynchronization underlies the human psychedelic state	Psylocibin	MEG	Theta	Decrease	Widespread
2016	Neural correlates of the LSD experience revealed by multimodal neuroimaging	LSD	MEG	Theta	Decrease	Widespread
2022	Low doses of LSD reduce broadband oscillatory power and modulate event-related potentials in healthy adults	LSD	EEG	Theta	Decrease	Widespread
2019	Neural correlates of the DMT experience assessed with multivariate EEG	DMT	EEG	Theta	Increase	Localized

The table presents a comparison of various studies examining the effects of psychedelic substances on brain activity. EEG (Electroencephalogram) and MEG (Magnetoencephalography) measures were utilized to assess changes in brain oscillatory bands, including Alpha, Beta, Delta, Gamma, and Theta. The 'Effect' column indicates whether the study reported an increase or decrease in the power of the specific band, while the 'Location' column denotes whether the effect was widespread across the brain or localized to specific regions.

## 5 Serotonin and surprise

The 5-HT system, comprising a complex network of neurons primarily located in the raphe nuclei on the midline of the brain stem, plays a pivotal role in various neural and cognitive processes, influencing both brain function and behavior. 5-HT, for example, participates in the regulation of mood (Dayan and Huys, 2009), sleep (Oikonomou et al., 2019), appetite (Lee and Clifton, 2010), memory (Teixeira et al., 2018) and decision-making (Homberg, 2012). The broad influence of the 5-HT system stems from its extensive projections across various cortical and subcortical regions. Despite the relatively small number of neurons expressing 5-HT, less than 0.1% (Okaty et al., 2019), projections from these neurons extensively innervate virtually all areas of the brain (Descarries et al., 2010). There are seven major classes of 5-HTRs, all with the exception of 5-HT_3_R, the only ionotropic receptor, are G-protein coupled receptors with complex intracellular chemical pathways (Mengod et al., 2010). The heterogeneous distributions of the various 5-HT receptor classes imply that 5-HT has the ability to regulate multiple aspects of animal cognition and behavior by modulating large-scale receptor networks across the entire brain (Salvan et al., 2023). 5-HTRs have been shown to modulate synaptic plasticity (Lesch and Waider, 2012, Li et al., 2021), neuronal excitability (Marek, 2010), and information processing (Teixeira et al., 2018). The 5-HT system, therefore, seems to fundamentally contribute to the intricate machinery underlying fundamental cognitive activities.

Beyond these multifaceted roles, it has been recently proposed that 5-HT might be responsible for the encoding of novelty and cognitive flexibility in dynamic environments (Matias et al., 2017, Clarke et al., 2004, Brigman et al., 2009, Grossman et al., 2022). To study the adaptation to an unexpected change in a familiar environment Matias et al. (2017) employed a reversal learning paradigm in mice in which the association between odor cues (conditioned stimuli, CSs) and different outcomes (unconditioned stimuli, USs), either a reward, a neutral stimuli or a punishment, were suddenly reversed during the execution of the task. Using photometric recordings in the dorsal raphe it was observed that when confronted with reward outcomes that deviated from expectations, whether they were better (positive prediction error) or worse (negative prediction error) than anticipated, 5-HT neurons displayed a similar pattern of transient excitation. There was a distinct activation upon the reversal with a neutral US stimulus. This response contrasts sharply with dopamine neurons, which exhibit an inhibitory response. This suggests that 5-HT neurons are responsive to unsigned violations of expectation in contrast to dopamine neurons that respond with either excitation or inhibition depending on the valence of the reward prediction error (Matias et al., 2017, Cohen et al., 2012, Dayan et al., 1997). In another study, tetrode recordings from optotagged 5-HT neurons showed that firing was positively correlated to unexpected uncertainty (i.e., surprise) in a dynamic foraging task in which mice are required to choose between two alternative sources of water (Grossman et al., 2022). Here unexpected uncertainty was defined as the deviation of the reward prediction error (RPE) from expected uncertainty, its historical weighted average. Interestingly, 5-HT neurons were found to correlate, both positively and negatively, also with expected uncertainty over relatively long periods (tens of seconds to minutes). Expected uncertainty, corresponding to precision, modulates the influence of prediction errors on updating priors, thereby adjusting the learning rate from current sensory information (Preuschoff et al., 2006, Preuschoff and Bossaerts, 2007, Nassar et al., 2012, Mcguire et al., 2014, Diederen and Schultz, 2015). These findings suggest a dual involvement of 5-HT in both first and second order predictions (Kanai et al., 2015).

In accordance with the proposed role of 5-HT in mediating prediction errors, impairments in performance were observed in a reversal learning task in marmosets with selective depletions of 5-HT in the prefrontal cortex (Clarke et al., 2004). The primary factor contributing to these impairments was the tendency to exhibit perseverative responding towards the stimulus that was previously rewarded. Further research indicated that this deficit specifically affected reversal learning and not attentional set shifting (Clarke et al., 2005). Additionally, it was discovered that the impairment in reversal learning was specifically associated with 5-HT depletion and not dopamine depletion in the orbitofrontal cortex. More specifically, selective blocking of 5-HT_2A_R significantly impaired reversal learning (Boulougouris et al., 2008, Furr et al., 2012), while low doses of LSD facilitated the same task (King et al., 1974, Kanen et al., 2022). Exposure to acute psilocybin was also observed to increase cognitive flexibility in a task requiring switching between previously learned strategies both in human and rodents (Doss et al., 2021, Torrado Pacheco et al., 2023).

The relation between prediction error and 5-HT was also investigated in human using fast-scan cyclic voltammetry in the dorsal striatum during a sequential investment game in which patients were instructed to select an investment level on each trial while real historical financial market prices unfolded (Moran et al., 2018). Here it was observed that 5-HT was significantly elevated in response to failed bets, indicating the occurrence of negative reward prediction errors (i.e., when participants predicted a reward that did not materialize). When categorizing the bets into high (wager >60% of current wealth) and low (wager <50% of current wealth), a particularly pronounced response was observed in relation to failed high bets. This outcome is linked to the stronger prediction of reward associated with high bets (high bets are logically associated with increased confidence of the prediction), leading to a more substantial error signal when the expected reward is not obtained. Notably, 5-HT exhibited a transient increase during instances of regret arising from not placing a substantial bet that would have been successful (i.e., positive prediction error, outcome better than expected). Interestingly, the 5-HT system did not show significant activation in response to predicted rewards or US, in line with prior research (Matias et al., 2017, Cohen et al., 2015, Liu et al., 2014).

Consistent with these findings, 5-HT neuromodulatory tone shows a tight relationship with pupil size (De Gee et al., 2021, Kloosterman et al., 2015, Friedman et al., 1973, Raisig et al., 2010), a behavioral marker providing an online surprise signal. Both in human and rodent, pupil size showed a remarkable increase upon experiencing prediction errors (Urai et al., 2017, Preuschoff et al., 2011, Harris et al., 2022, Jordan and Keller, 2023, Filipowicz et al., 2020). Moreover, the association between 5-HT and pupil size is modulated by the amount of uncertainty in the environment, representing the baseline amount of prediction errors. In mice, in situations of low uncertainty, optogenetic stimulation of the 5-HT system consistently leads to significant transient pupil dilations. However, when the environment is already uncertain, this effect is diminished (Cazettes et al., 2021). This finding resembles precision weighting, the mechanism that facilitates robust learning signals to drive more substantial hypothesis revision. Conversely, in conditions of heightened uncertainty, the prediction error unit are inhibited, diminishing its impact on the updating of priors (Auksztulewicz and Friston, 2016).

Additionally, 5-HT seems to play a direct role in perception. Experimental data from human recordings using fast-scan cyclic voltammetry showed that sub-second signaling of serotonin in the human striatum is involved in real-time inference about the external world. Bang et al. (2020) showed that there is a transient increase in 5-HT levels when coherence in a dot motion paradigm, the fraction of coherently moving dots, is low. Coherence here corresponds to sensory uncertainty. In contrast, there is a transient decrease in 5-HT levels when coherence is high. These observations suggest that 5-HT dynamically tracks the level of uncertainty present in sensory information, displaying opposite modulation depending on the degree of coherence.

Collectively, these findings present a captivating portrayal of the involvement of 5-HT in monitoring novelty across multiple levels of abstraction. 5-HT appears to function as a signal for prediction errors and uncertainty, with a possible direct link with perception. Intriguingly, the involvement of 5-HT in indexing uncertainty can be used to explain its known effect on behavioral inhibition (Soubrié, 1986) and patience (Miyazaki et al., 2014, Miyazaki et al., 2011, Li et al., 2016). Using optogenetics it was shown that high reward probability maximizes the ability of 5-HT to promote patience (Miyazaki et al., 2014). This result was explained using a Bayesian decision model, suggesting that 5-HT neuron activation increases the subjective confidence of reward delivery. In other terms, we can consider trials with high reward probability to engender a clear expectation of reward. Stimulating 5-HT release and consequently, according to our model, increasing uncertainty in a context where a clear expectation is present (high precision afforded to the prior) can cause a discounting of sensory evidence in favor of the expectation. Moreover, uncertainty in the sensory data, manipulated in the study by changing the reward timing, also increases waiting times, consistent with a further strengthening of the precision associated to the prior.

The activation of the 5-HT system is likely to initiate a cascade of effects, engaging various classes of receptors that, in turn, may influence other neuromodulatory pathways. For example, photostimulation of the raphe nuclei influences pupil size with a longer latency compared to noradrenergic stimulation, suggesting an indirect effect (Cazettes et al., 2021, Yu et al., 2004). Additionally, 5-HT neurons can directly modulate dopaminergic neurons (Chang et al., 2021, Beier et al., 2015). These intricate interactions highlight the complex nature of the downstream effects of 5-HT.

## 6 The psychedelic experience as synthetic surprise

At a more abstract level, yet fundamentally anchored in a predictive coding paradigm, we can employ the concept of cognitive penetrability to elucidate the psychedelic experience. The central idea of our proposal is inspired by the aforementioned experimental data pointing at the role of 5-HT in signaling prediction error and it can be succinctly conveyed by a simple thought experiment. Let us assume the existence of some chemical compounds able to trigger, in a specific and compartmentalized fashion, one emotional state. According to the cognitive penetrability and affective realism hypotheses, such compounds will potentially have a direct effect on sensory perception and cognition. Consequently, the deliberate artificial induction of fear is expected to impact prior beliefs. This effect can be expected to result in ambiguous stimuli being perceived as more alarming in comparison to a baseline neutral state. If we were to elicit instead anger, we can hypothesize that the subject would be biased towards inferencing a threat in the presence of an ambiguous stimulus. What if we artificially elicited surprise? Probably everything would look surprising even in absence of any objective novelty in the environment. Moreover, while other emotions can be expected to have an influence particularly in ambiguous contexts, an artificially imposed surprise affect would directly increase the uncertainty of the environment. This can inherently increase the perception of illusory patterns (Whitson and Galinsky, 2008). Cognitive penetrability tend to occur when an input is uncertain or ambiguous, especially when a higher level representation can successfully explain away the prediction error (Hohwy, 2013a). Moreover, hallucinations and delusions have been reported to be influenced by the emotional state of the subject (Freeman and Garety, 2003, Smith et al., 2006, Freeman et al., 2013). We propose that this is what happens upon consumption of psychedelic compounds: a synthetic surprise affect enforced on our inferential machine and cognition.

An interesting corollary, deriving from the hypothesis that psychedelics are first and foremost (Bayesian) surprise-inducing compounds, is that the perceptual alterations are to be considered downhill effects of the overactivation of prediction error signaling. It follows that the sense of profound surprise, also described as awe (the emotional response to something perceived as extraordinary), often reported upon consumption of psychedelics (Preller and Vollenweider, 2018), is not, as intuitively assumed, triggered at least in part by sensory alterations (Aqil and Roseman, 2023, Shiota et al., 2007, Chen and Mongrain, 2021) but, on the other hand, is the upstream cause of those alterations. The awe and novelty experienced under psychedelics are proposed to be the primary driver, setting off a cascade of perceptual changes, rather than a result of these changes.

In a normal state, surprise arises from the interplay of sensory inputs and internally generated predictions. Its magnitude is directly proportional to the extent of disparity between predictions and actual sensory input and is associated with physiological changes, including an increased heart rate, elevated blood pressure (Boiten, 1996, Ekman et al., 1983, Epstein et al., 1975, Jang et al., 2015), heightened skin conductance response (Alaoui-Ismaïli et al., 1997, Levenson et al., 1990), and enlarged pupil size (Kloosterman et al., 2015, Lavin et al., 2014). Studies indicate that substances such as LSD and psilocybin can trigger these identical physiological responses. Specifically, LSD and psilocybin have been observed to increase blood pressure, heart rate, and pupil size (Holze et al., 2022, Schmid et al., 2015). Additionally, LSD has been found to elicit a clear skin conductance response (Greiner et al., 1958). These findings suggest that the psychedelic experience evokes an interoceptive state resembling the one elicited by natural surprise. Following both historical and contemporary theories of emotion (James, 1884, Seth, 2013), which emphasize the integral role of physiological responses in shaping our emotional experiences, we propose that this altered interoceptive state might have an important emotional influence.

The psychedelic experience itself is so foreign compared to the normal palette of conscious states that it seems to naturally require an extraordinary explanation, a fundamental change in brain function. This assumption is likely driven by the inherent novelty of the psychedelic experience, activating brain mechanisms that are usually triggered by unexpected stimuli in normal physiological conditions. Consequently, according to our model no fundamental alteration of brain functioning should be observed. The psychedelic state should, instead, bear resemblance to a native surprised state. In accordance with this view the one neural signature common to all psychedelics, a reduction in alpha power, is also observed naturally in result of exposure to novelty. Using multilaminar electrodes in monkey during a delayed match to sample task, alpha power of cortical areas was decreased when the task was less predictable (Bastos et al., 2020). In humans EEG recordings during a novelty detection task showed that alpha power was decreased upon the detection of a temporary unpredictable stimulus (Busch et al., 2009, Scheeringa et al., 2011, Arana et al., 2022, Bacigalupo and Luck, 2022). Furthermore, the anterior cingulate cortex (ACC), a region implicated in encoding surprise (Hayden et al., 2011, Vassena et al., 2020), showed increased spiking and altered neuronal ensemble activity in mice in response to psilocybin (Golden and Chadderton, 2022).

A state of enhanced surprise has been previously associated with schizophrenia (Adams et al., 2013). Trait abnormalities in these patients have been linked to difficulties in accurately predicting sensory input, leading to a state where all perceptions are experienced as surprising. This has downstream effects on event-related potentials (ERP) measurable via electrophysiological methods. The mismatch negativity (MMN) is an ERP component primarily elicited in response to rare or unexpected stimuli in a sequence of standard stimuli. However, in schizophrenic patients, the MMN responses to such oddball stimuli are diminished (Winterer and Weinberger, 2004). The predictive coding framework offers an explanation for these deficits (Fletcher and Frith, 2009). This framework posits that the fundamental issue in schizophrenia might not be the prediction of sensory input per se, but rather lies in the balance between the precision assigned to prior beliefs and sensory evidence (Friston, 2005, Corlett et al., 2011). Simulations can be employed to demonstrate how abnormal levels of sensory precision, whether increased or attenuated, can account for atypical responses to surprising events (Adams et al., 2013).

In accordance with our hypothesis, some reports showed that psychedelics induced alterations in ERP responses that are in line with a state of surprisal. Specifically, both LSD and psilocybin could reduce significantly the MMN amplitude in response to deviant stimuli in human (Duerler et al., 2022, Timmermann et al., 2018). Other studies, however, have failed to observe differences (Umbricht et al., 2003, Schmidt et al., 2012).

In considering the consequences of inducing a state of synthetic surprise, that is an increase in prediction error and uncertainty, within the predictive coding framework, we can explore the possible outcomes and examine their alignment to the observed effects of psychedelics. These outcomes are shaped by the precision associated to the prediction error. Precision is the inverse of the expected uncertainty (Feldman and Friston, 2010) and can be modeled as the weighted historical average of the prediction errors (Grossman et al., 2022). A consistently enhanced prediction error is expected to increase the expected uncertainty, therefore, reducing the precision afforded to sensory data. The complexity of the situation, however, is further compounded when considering the roles of other neuromodulator in precision weighting. Specifically, in addition to 5-HT, precision weighting has been linked also to acetylcholine and dopamine (Feldman and Friston, 2010, Yu and Dayan, 2005, Moran et al., 2013, Pérez-González et al., 2023, Haarsma et al., 2021, Grossman et al., 2022, Soltani and Izquierdo, 2019). It is conceivable that the precision of the prediction error (PE) might be enhanced via 5-HT independent routes. Precision is anticipated to dynamically affect information processing as follows:

1. High PE precision → Enhanced belief updating: artificially increasing the prediction error would lead to more substantial updates in beliefs, allowing for greater flexibility in incorporating new information and revising prior assumptions.
2. Low PE precision → Strong priors: the brain relies more heavily on existing beliefs due to the reduced reliability of sensory input. This could result in a tendency to perceive and interpret experiences in accordance with pre-existing expectations, possibly leading to perceptual biases or hallucinations.

Considering the enhanced belief updating, the synthetic surprise model can explain the heightened openness, ego dissolution and some psychosis like symptoms observed in subjects under the influence of psychedelics (Maclean et al., 2011, Carhart-Harris et al., 2016a, Letheby and Gerrans, 2017, Nour et al., 2016). In a normal state of consciousness, our awareness is limited to a specific set of accessible priors that aid in explaining incoming sensory information. These priors are continuously updated to align with the sensory input, with each prediction error signaling the need for a new version (prior n → prior n + 1) to be considered. At a theoretical onset of perception, each prior can be associated with a likelihood, and when a prediction error signal reaches the higher layers of processing, new priors are sequentially explored until the most suitable one is identified. Under the influence of psychedelics, the heightened activation of prediction error signals pushes the brain into a more malleable state. This plastic state can access priors that would typically be inaccessible in a normal state. Interestingly, a similar mechanism has been previously proposed to explain the genesis of delusions (Fletcher and Frith, 2009, Hemsley and Garety, 1986). This expanded range of accessible priors, upon a persistent prediction error signal, contributes to an increased openness to external ideas and concepts, even those that may appear implausible in a default state. Consistent with this idea, both psilocybin and LSD have been found to enhance the accessibility of remote associations (Family et al., 2016, Spitzer et al., 1996, Kuypers et al., 2016), thereby facilitating the activation of cognitive contents that typically remain latent under normal circumstances. This suggests that psychedelics promote a broader range of cognitive associations and allow for the exploration of novel connections and perspectives. The increased number of accessible priors might underlie the noetic quality characteristic of psychedelic experiences, the profound sensation of accessing direct insights and revelations (Pahnke and Richards, 1970, Timmermann et al., 2022). Such moments of insight are believed to play a foundational role in how psychedelics reshape beliefs in pathological situations (Garcia-Romeu et al., 2019, Carhart-Harris et al., 2018, Davis et al., 2020, Williams et al., 2021, Noorani et al., 2018).

Strong priors, on the other hand, can explain the hallucinations typical of the psychedelic experience (Corlett et al., 2019, Kwan et al., 2022). In this context hallucinations, defined as perceptions without identifiable external stimuli, are not mere errors in sensory processing but rather a result of the predictive machinery operating on overly precise priors. It is posited that hallucinations arise when priors excessively influence sensory interpretation, overshadowing actual sensory evidence. This theory can explain how conditioned hallucinations can be elicited by associating stimuli of different modality, a long known phenomenon observed in various studies (Powers et al., 2017, Ellson, 1941, Powers et al., 2016, Seashore, 1895, Barber and Calverley, 1964).

## 7 Possible biological implementations of the synthetic surprise model

Although many aspects of the implementation of the predictive coding hypothesis are still being investigated, recent years have seen progress in unraveling the underlying circuitry. Within this framework, prediction errors play a fundamental role. They serve as crucial signals that trigger updates in the internal models, allowing the brain to refine its predictions and minimize the mismatch between expectations and reality. Assuming the existence of prediction error neurons (Rao and Ballard, 1999), their fundamental function would involve comparing top-down inputs, which provide predictions of sensory input, with bottom-up sensory-driven inputs in order to discern the difference. One potential mechanism for achieving this comparison is through a subtractive process, which would entail prediction error neurons exhibiting balanced and opposing weights for the two types of input (Rao and Ballard, 1999, Keller and Mrsic-Flogel, 2018). Consequently, if the bottom-up sensory input is excitatory, the influence of the top-down input should be inhibitory, and vice versa. This bidirectional interaction ensures a dynamic interplay between top-down predictions and bottom-up sensory signals within prediction error neurons. The predictive processing framework entails two distinct types of prediction error neurons: positive and negative. Positive prediction error neurons perform the subtraction of top-down predictions from sensory input, while negative prediction error neurons subtract sensory input from top-down predictions (Keller and Mrsic-Flogel, 2018, Rao and Ballard, 1999). If the relative strengths of top-down and bottom-up inputs are opposing within individual neurons, any temporary imbalance between these two sources would give rise to prediction error responses.

**Fig. 2:**
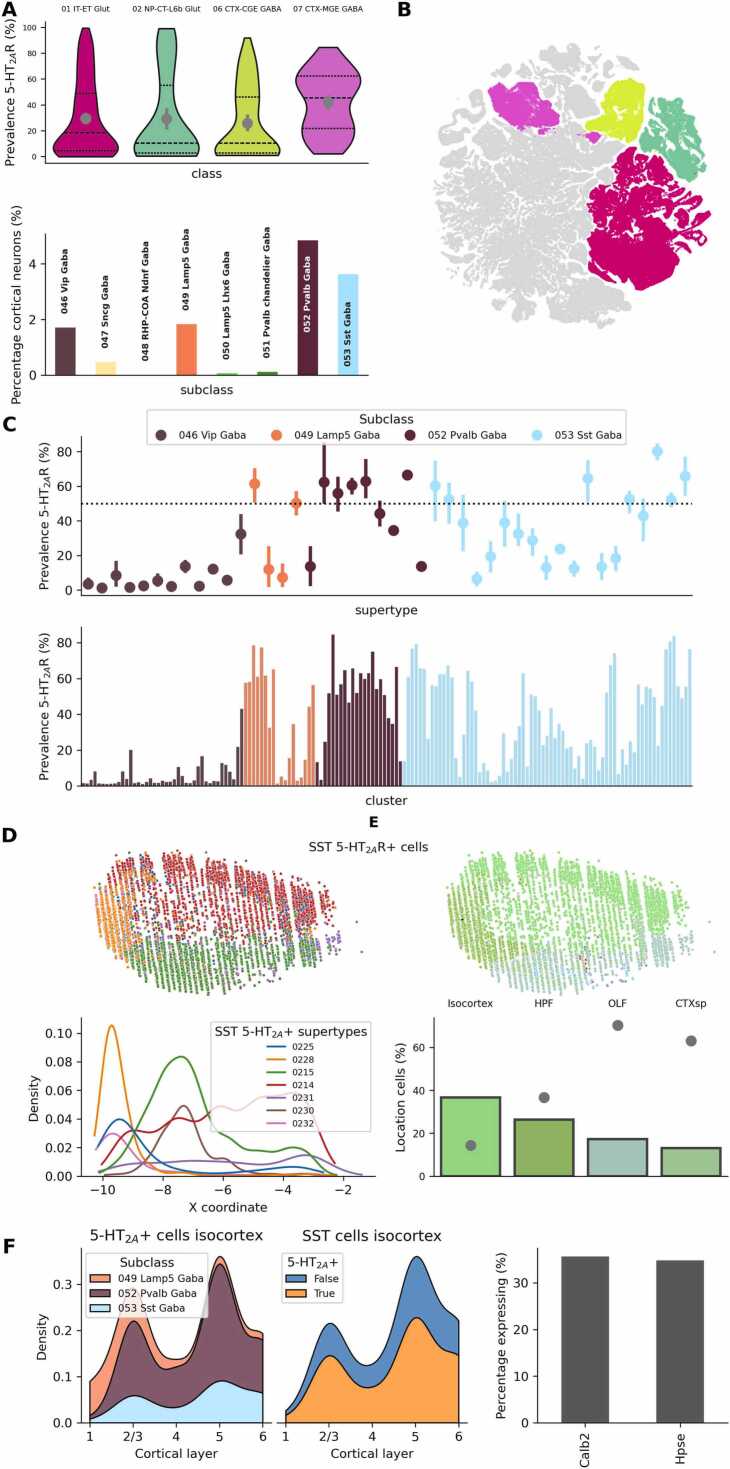
Analysis of 5-HT_2A_R RNA transcription in mouse cortical neurons. A Top: average prevalence of 5-HT_2A_R RNA in cortical clusters grouped by class. 07 CTX-MGE GABA contains a significant number of clusters that express high levels of 5-HT_2A_R RNA. Bottom: Size of isocortex inhibitory subclasses expressed as percentage over the total amount of isocortex neurons. Isocortex inhibitory neurons are contained in the classes 06 CTX-CGE GABA and 07 CTX-MGE GABA. Groups are color-coded using to the original atlas data. B UMAP representation of all cell types. Cortical classes are color-coded according to A. C Top: Prevalence of 5-HT_2A_R RNA for each supertype belonging to the main isocortex inhibitory subclasses. Lamp5, PV and SST show highly enriched supertypes (>50%). Bottom: Same as top but grouped by cluster. Dotted line indicates 50% threshold. D Top: spatial location (lateral view) of cells belonging to SST 5-HT_2A_R+ supertypes. Bottom: distribution of cells plotted above along the x coordinate. Supertype numbers are visible in the legend. E Top: same as D but color-coded according to anatomical division (Isocortex, HPF: hippocampus, OLF: olfactory cortex, CTXsp: cortical subplate). Bottom: bars represent the spatial distribution of cells in SST 5-HT_2A_R+ supertypes. Dots represent the fraction of SST cells per anatomical division belonging to 5-HT_2A_R+ supertypes. Most of SST cells in OLF and CTXsp show 5-HT_2A_R RNA expression. **F** Distribution of isocortex 5-HT_2A_R+ neurons across layers and main inhibitory subclasses (left). Distribution of isocortex SST 5-HT_2A_R positive and negative neurons across layers (center). Co-expression of Calb2 and Hpse in Isocortex SST 5-HT_2A_R+ neurons(right).

The suggested mechanism explaining the impact of psychedelics on predictive coding circuitry, though speculative, is based on two recent lines of experimental evidence and an analysis of recent transcriptomic data, which support the presence of 5-HT_2A_Rs in SST interneurons. First, studies have underscored the significant role played by SST inhibitory interneurons in computing prediction errors (Attinger et al., 2017, Green et al., 2023). In the context of expected visual flow resulting from locomotion, it was shown that SST interneurons in V1 exert inhibitory control over neurons responsible for detecting negative prediction errors (Attinger et al., 2017). These negative prediction errors occur when the expected visual flow, anticipated because of self-initiated locomotion, fails to materialize. Moreover, Sst44 positive SST neurons in the mouse parietal cortex provide an error signal in goal-directed navigation (Green et al., 2023) and a subgroup of SST interneurons in mice was also showed to be activated by novelty using 2-photon calcium imaging (Garrett et al., 2023). Second, there is evidence pointing to the possible activation of SST interneurons by 5-HT_2A_Rs. While numerous studies have observed responses to 5-HT_2A_R activation consistent with the stimulation of inhibitory cells (as discussed in the Introduction), only a few have precisely identified the class of interneurons involved. Notably, a recent study focused on the mouse entorhinal cortex found that SST interneurons are activated by 5-HT, specifically through the activation of 5-HT_2A_R (De Filippo et al., 2021). More data is necessary to establish whether SST interneurons in other brain areas are also activates by 5-HT_2A_R. Third, analysis of the mouse whole-brain transcriptomics cell type atlas provided the Allen Institute for Brain Science (Yao et al., 2023), showed enrichment of 5-HT_2A_R RNA in SST cortical neurons. The atlas categorizes neurons in a hierarchical system (cell>cluster>supertype>subclass>class). Four classes account for the vast majority of neurons present in the isocortex (99.62%): 01 IT-ET Glut, 02 NP-CT-L6b Glut, 06 CTX-CGE GABA and 07 CTX-MGE GABA. Medial ganglionic eminence (MGE)-derived GABAergic neurons, including SST interneurons, showed the higher percentage of clusters showing significant enrichment in 5-HT_2A_R RNA (Fig. 2A-B). A cell was considered to express 5-HT_2A_R RNA if log(CPM)> 3.5 (CPM, counts per million reads), the same stringent threshold used in the original report to classify by neurotransmitter type (Yao et al., 2023). We focused on the main cortical inhibitory subclasses by number of cells: Vip, Lamp5, PV and SST (Fig. 2A). Except for Vip neurons, significant 5-HT_2A_R RNA expression was observed in supertypes of all other subclasses. A 5-HT_2A_R enriched supertype (5-HT_2A_R+) was defined as having at least 50% 5-HT_2A_R prevalence, i.e. half of the cells belonging to the supertype express 5-HT_2A_R RNA. SST 5-HT_2A_R+ supertypes were distributed across the entire cortex (Fig. 2D) with a majority of SST 5-HT_2A_R+ cells present in the isocortex (Fig. 2E). 5-HT_2A_R+ cells were present in all cortical layers (Fig. 2F). L1 shows high proportion of Lamp5 5-HT_2A_R+ cells. PV and SST 5-HT_2A_R+ cells are present across all other layers, particularly prominently in L2/3 and L5. A stable fraction of SST interneurons across layers expressed 5-HT_2A_R RNA (63.76 ± 0.98%). Sst44, the marker used to identify SST interneurons involved in error detection during navigation, was found in a significant number (≈40%) of *Calb2+* and *Hpse+* neurons (Green et al., 2023). Notably, cortical SST 5-HT_2A_R+ were also found to express a substantial amount of these two genes (*Calb2*:35.68%, *Hpse:34.80%*, Fig. 2F right). We also provide an online visualization tool for the exploration of 5-HT_2A_R RNA expression based on the MERFISH dataset of (Yao et al., 2023).

Considering the variations in 5-HT levels across the sleep-wake cycle (Oikonomou et al., 2019), it is reasonable to speculate that the activation of SST interneurons might be influenced by these fluctuations. Hence, we would anticipate a positive correlation between the spiking activity of SST interneurons and the levels of 5-HT across different states of the sleep-wake cycle. Specifically, we would expect the highest activation of SST interneurons during wakefulness, lower during slow-wave sleep, and lowest during rapid eye movement (REM) sleep. Supporting this notion, a study that directly measured the activity of various neuronal classes across different states found that SST interneurons in the dorsal cortical surface exhibited exactly the expected pattern of activation (Niethard et al., 2016). Notably, this pattern was not observed in PV interneurons, which displayed similar levels of activation during both REM sleep and wakefulness. These findings align with a positive correlation between the activity of SST interneurons and 5-HT release.

**Fig. 3:**
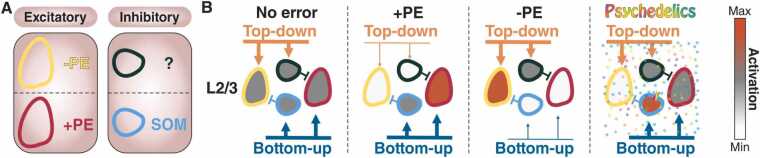
Synthetic surprise implementation via SST interneurons activation. A Components of the prediction error (PE) computation circuit. B In situations of positive (+) PE, such as experiencing visual flow in the absence of locomotion, the bottom-up sensory information exceeds predictions. This discrepancy leads to the activation of SST neurons, which in turn inhibit the neurons encoding negative (-) PE. Conversely, negative PEs arise when top-down predictions fail to align with the expected sensory input, such as experiencing a visual flow freeze during active locomotion. We hypothesize that psychedelics may activate SST interneurons responsible for inhibiting negative PE neurons. This could result in an unbalanced positive PE reaching deeper layer pyramidal cells, which encode priors.

Within the previously described predictive coding circuitry, it is hypothesized that the prolonged activation of SST interneurons mediated by 5-HT_2A_Rs leads to a paradoxical over-inhibition of negative prediction error neurons. Experimental data shows that L2/3 excitatory neurons, supposed to compute prediction errors, react to visuomotor mismatches exhibiting membrane potential responses ranging from strongly depolarizing to strongly hyperpolarizing (Jordan and Keller, 2020). A blanket SST interneurons activation by 5-HT_2A_Rs is expected to influence also L2/3 neurons that physiologically would receive only weak inhibitory input. Consequently, a discrepancy arises between the signaling of positive and negative prediction errors, even in the absence of a significant mismatch with sensory input, ultimately resulting in an unbalanced positive prediction error signal (Fig. 3). In the context of locomotion, a positive prediction error is triggered when an unexpected visual flow is experienced in the absence of self-generated movement. Intriguingly, this description resonates with commonly reported visual distortions induced by psychedelics, such as the perception of movement in stationary objects (Muthukumaraswamy et al., 2013, Schmid et al., 2015).

Illustration modified from Padamsey and Rochefort (2020).

Other circuit implementation of the predictive coding hypothesis exists, for example predictions can be theoretically implemented via lateral inhibition instead of feedback connections (Spratling, 2017, Huang and Rao, 2011, Olshausen and Field, 1997), in another variant the entire computation can be performed by local voltage dynamics in individual neurons (Mikulasch et al., 2023). In both cases SST interneurons, being involved in lateral inhibition (Adesnik et al., 2012) and influencing dendritic compartments of excitatory cells (Rudy et al., 2011), are expected to have major impact. Furthermore, we cannot disregard the possibility of additional direct effects in primary cortices, potentially involving a modification of surround suppression within the cortex through the activation of SST interneurons (Michaiel et al., 2019). In exploring the effects of psychedelics through the lens of the predictive coding framework, we recognize that our hypothesis is constrained by the current limited understanding of the biological mechanisms underlying this framework. In accordance with these limitations, we concentrate on SST neurons, as emerging evidence suggests their involvement in prediction error signaling (Attinger et al., 2017, Green et al., 2023). It's important to note, however, that 5-HT_2A_Rs potentially influence a broad spectrum of neuronal classes. For instance, cortical Lamp5 and PV neurons exhibit a high concentration of 5-HT_2A_R RNA (Fig. 2). This is supported by studies in mice showing that PV neurons can be activated by 5-HT_2A_R (Puig et al., 2010, Athilingam et al., 2017). Some excitatory subclasses also show considerable expression, subclasses localized in the claustrum (001 CLA-EPd-CTX Car3 Glut), L5 (005 L5 IT CTX Glut, 006 L4/5 IT CTX Glut), L6 (004 L6 IT CTX Glut) and L6b (027 L6b EPd Glut, 028 L6b/CT ENT Glut, 029 6b CTX Glut) exhibit a prevalence of > 50% (see online visualizer). Another complication not addressed in this review is the functional selectivity of 5-HT_2A_Rs (Gonzalez-Maeso et al., 2007, Lopez-Gimenez and Gonzalez-Maeso, 2017).

## 8 Implications for the clinical use of psychedelics

In recent years, there has been a resurgence of interest in re-evaluating the therapeutic potential of psychedelics as groundbreaking treatments for psychiatric disorders (Mcclure-Begley and Roth, 2022, Goodwin et al., 2022). While this concept may seem novel, it builds upon earlier explorations conducted during the 1950 s and 1960 s, when psychedelics were investigated as potential therapies for depression and alcoholism (Abramson, 1966). Recent experimental results are encouraging towards a potential role for psychedelics in clinical settings, specifically for the treatment of depression and anxiety-related disorders (Schimmers et al., 2023, Holze et al., 2023, Carhart-Harris et al., 2021, Daws et al., 2022, Heal et al., 2023). Interestingly, the therapeutic effect of psilocybin, in humans, has been found to correlate with various subjective qualities of the psychedelic experience (Roseman et al., 2018, Barrett and Griffiths, 2018, Griffiths et al., 2016, Aaronson et al., 2023), pointing at the importance of the subjective experience. It is worth mentioning, however, that some studies performed in rodents, have presented contrasting findings (Hesselgrave et al., 2021, Moliner et al., 2023, Kaplan et al., 2022, Cao et al., 2022, Cameron et al., 2021, Dong et al., 2021, Dunlap et al., 2020). This divergence highlights the necessity for additional research in this field, particularly considering that animal models of major depression display substantial shortcomings in fully replicating the human condition (Planchez et al., 2019, Belzung, 2014).

The clinical use of psychedelics presents a fascinating avenue for exploring the implications of the synthetic surprise model. The predictive coding framework offers valuable insights into the underlying mechanisms of depression, emphasizing the role of excessively strong and narrow predictions within specific neural networks (Barrett et al., 2016). This framework suggests that depression arises from a "locked-in" brain state, characterized by an insensitivity to prediction errors (Barrett et al., 2016, Carhart-Harris, 2019). This state hinders the ability of the brain to update its internal models, leading to a breakdown in adaptive processing (Barrett et al., 2016). The model proposes that individuals with depression have a propensity to predominantly expect negative events or experiences, which they subjectively validate by reappraising disconfirming evidence, ultimately leading to the establishment of a self-reinforcing negative feedback loop. The underlying cause can be identified in the excessive precision assigned to prior beliefs with negative valence as a key contributor to the pathology, accompanied by a decrease in prediction errors (Kube et al., 2020). Intriguingly, a theoretical compound that enhances prediction errors seems to logically be a fitting candidate for therapeutical interventions in such context.

According to our hypothesis psychedelics fulfill exactly this role by enforcing a synthetic surprise affect. We propose that the clinical value of psychedelics might be strictly related to their surprise-inducing property. By extending this logic, other psychoactive compounds might also offer beneficial effects. In fact, when individuals encounter for the first time the effects of a psychoactive substance, they are likely to be surprised. Accordingly, throughout history a remarkable spectrum of substances have showcased promising therapeutic outcomes: anxiolytics like benzodiazepines (Ogawa et al., 2019) and barbiturates (Horwitz and Rubin, 2023), empathogens like MDMA (Vermetten and Yehuda, 2020), psychostimulants like methylphenidate (Houlihan, 2011, Aga-Mizrachi et al., 2014, Daly, 2000), theophylline (Murphy et al., 1980), modafinil (Price and Taylor, 2005) and cocaine (Freud, 1973, Heimann, 1887, Post et al., 1974), depressants like opioids (Emrich et al., 1982, Carlson and Simpson, 1963), esmethadone (Fava et al., 2022), GHB (Bosch et al., 2012) and cannabis (Sachedina et al., 2022, Martin et al., 2021), dissociatives like ketamine (Anand et al., 2023, Marwaha et al., 2023, Marcantoni et al., 2020), Dextromethorphan (Tabuteau et al., 2022), nitrous oxide (Varias et al., 2020, Nagele et al., 2015, Nagele et al., 2021), Salvia divinorum (Taylor and Manzella, 2016), and methoxetamine (Striebel et al., 2017), deliriants like scopolamine (Furey and Drevets, 2006, Drevets and Furey, 2010) and also anesthetics like propofol (Theresa et al., 2023, Mickey et al., 2018), sevofluorane (Zhang et al., 2016, Guo et al., 2019, Chen et al., 2015) and isofluorane (Theresa et al., 2023, Antila et al., 2017, Weeks et al., 2013). Notably, these compounds affect virtually all known existing neurotransmitter systems, including acetylcholine, dopamine, GABA, noradrenaline, adenosine, 5-HT, glutamate, GHB, sigma, opioid, and histamine receptors. Particularly puzzling is the recent report of an absence of difference between general anesthesia and ketamine for the treatment of depression in a triple-masked, randomized, human placebo-controlled trial (Lii et al., 2023).

We propose that the potential antidepressant properties of these compounds may not be attributed primarily to a specific pharmacological action, but rather to the subjective experience of encountering something profoundly novel, a phenomenon achievable through alterations in various neuromodulatory pathways. Despite the promising results seen with the aforementioned array of compounds, we are still in search of a truly effective pharmacological solution to depression. One common limitation predicted by our hypothesis is that upon repeated administrations, the novelty effect is expected to wane. In contrast, psychedelics are likely to have a more enduring effect due to their intrinsic ability to directly induce surprise. Nonetheless, the possibility of negative outcomes due to intake of psychedelics should still be kept in mind (Bienemann et al., 2020, Kopra et al., 2022, Johnson et al., 2008, Simonsson et al., 2023).

## 9 Outlook

It is of interest to find an experimental paradigm able to discern the validity of our model. It would be useful to determine whether under psychedelics priors gain the capacity to shape perception, aligning with the strong prior theory and the synthetic surprise model. The tasks developed previously by Powers et al. (2017) and Schmack et al. (2021), associated with psychedelics could offer valuable glimpses into the interplay between priors and perception under the effect of psychedelics.

If the activation of 5-HT_2A_R truly plays a role in indexing uncertainty and signaling prediction error, its antagonism is expected to trigger notable psychological alterations. Initial evidence points in this direction, with some data highlighting its involvement in reversal learning in rodents. Prediction errors are essential for the successful execution of a reversal learning task. In line with our hypothesis 5-HT_2A_R antagonism significantly increases the number of trials necessary to learn the reversal association, causing a pattern of perseverative responding (Boulougouris et al., 2008, Furr et al., 2012). Interestingly, applying psilocybin in conjunction with ketanserin was showed to further reduce vigilance compared to psilocybin alone. Moreover, blocking 5-HT_2A_R did not rescue performance deficiencies in attention induced by psilocybin (Carter et al., 2005). It is essential to further investigate this topic in humans to achieve a deeper understanding of the role of 5-HT_2A_R in decision-making and cognitive flexibility.

Regarding the circuit implementation, it is necessary to precisely map the effects of 5-HT_2A_R activation across different classes of excitatory and inhibitory neurons across different cortical areas. While recent data have shown that SST interneurons are activated by 5-HT_2A_Rs in the entorhinal cortex (De Filippo et al., 2021), it remains an open question whether this specific class of interneurons is activated by 5-HT_2A_R across the cortex at a broader scale. Analysis of transcriptomic data points in this direction (Fig. 2). By systematically mapping the impact of 5-HT_2A_Rs activation on diverse neuronal populations within the cortex, we can gain a more comprehensive understanding of the underlying neural mechanisms that mediate the effects of psychedelics and their relevance in shaping cortical activity and cognitive functions.

If the therapeutic benefits of psychedelics truly hinge on their ability to induce surprise, it suggests that other interventions inducing unexpected uncertainty might similarly yield positive effects. This hypothesis prompts a deeper exploration into non-pharmacological methods that can elicit similar states.

## 10 Conclusion

We propose that psychedelics exert their influence by inducing a state of synthetic surprise, achieved through the artificial generation of prediction error. Foundation of this model is the proposed role of 5-HT in signaling prediction error. The downstream effect of this increase in prediction error depends on its precision weighting, a process likely mediated by various neuromodulators. In this way, priors can dynamically be strengthened or weakened. Within this model, hallucinations can be explained by the strong prior hypothesis. By incorporating recent discoveries about 5-HT and predictive coding, we offer a comprehensive perspective on how these substances profoundly alter perception. More experiments are necessary to test the proposed circuit implementation based on activation of interneurons by 5-HT_2A_Rs. Nevertheless, the synthetic surprise hypothesis puts forth a falsifiable theory that strives to elucidate the effects of these fascinating substances.

## CRediT authorship contribution statement

**Roberto De Filippo**: Conceptualization, Writing – original draft, Writing – review & editing, Visualization. **Dietmar Schmitz**: Funding acquisition, Writing – review & editing.
